# Supplementary material for: Automated Quantitative Image-Derived Input Function for the Estimation of Cerebral Blood Flow Using Oxygen-15-Labelled Water on a Long-Axial Field-of-View PET/CT Scanner
Source: Diagnostics (Basel). 2024 Jul 24;14(15):1590. doi: 10.3390/diagnostics14151590 (PMC11311987; doi:10.3390/diagnostics14151590)
Supplement: Supplementary file 1 [file diagnostics-14-01590-s001.zip › diagnostics-3098964-supplementary.pdf]

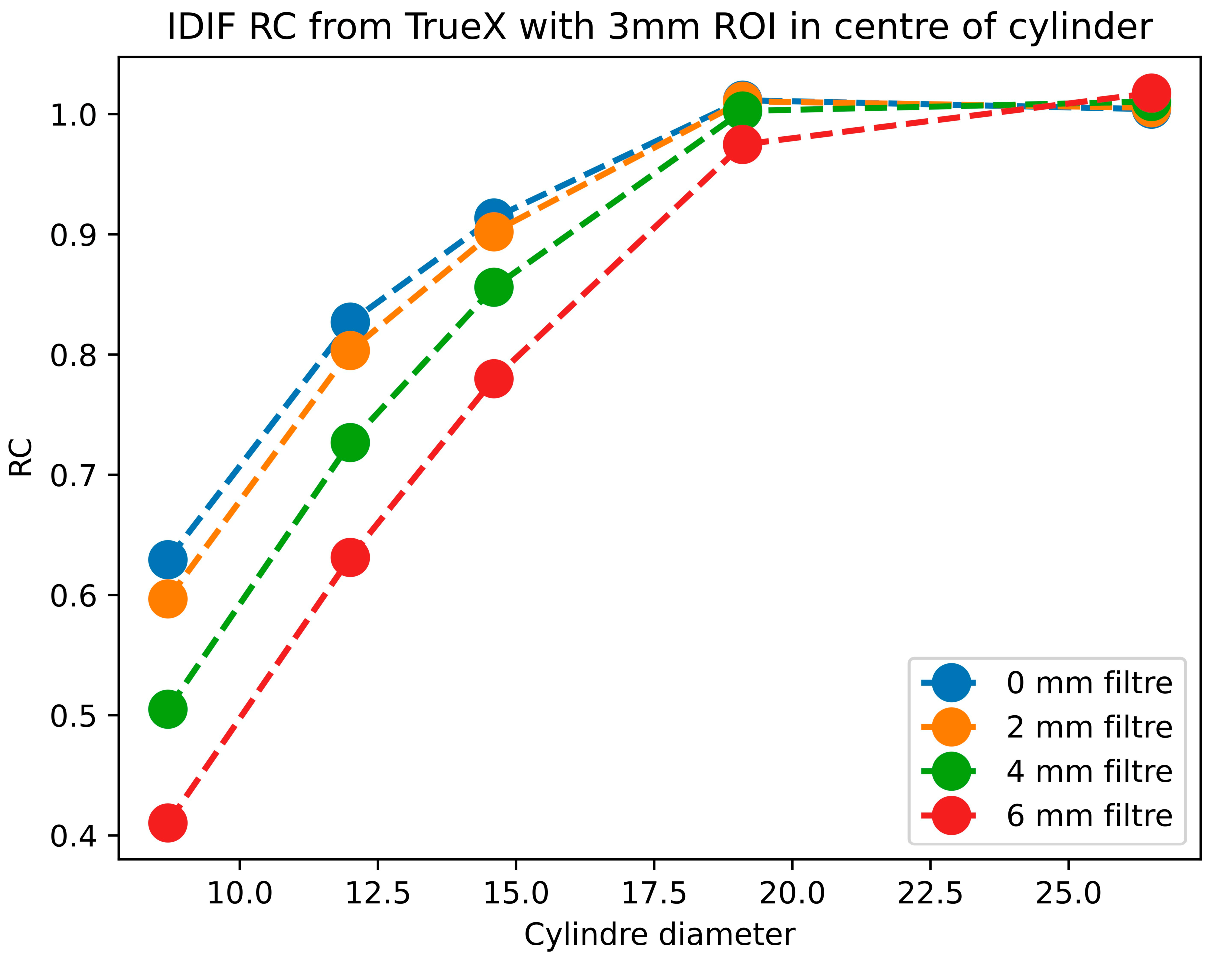

Figure S1. Recovery coefficients (RC) of the [^{15}O]-H_2O phantom from a 3 mm ROI with difference post reconstruction Gaussian filters. For typical aortic diameters of 25 mm or more the recovery coefficient is 1.
